# (3*RS*,4*SR*)-Methyl 4-(2-chloro-5,8-di­methoxy­quinolin-3-yl)-1-phenyl­pyrrolidine-3-carboxyl­ate

**DOI:** 10.1107/S1600536808031838

**Published:** 2008-10-09

**Authors:** Saida Benzerka, Abdelmalek Bouraiou, Sofiane Bouacida, Salah Rhouati, Ali Belfaitah

**Affiliations:** a Laboratoire des Produits Naturels, d’Origine Végétale et de Synthèse Organique, PHYSYNOR, Université Mentouri–Constantine, 25000 Constantine, Algeria; b Département de Chimie, Faculté des Sciences et Sciences de l’Ingénieur, Université A. Mira de Béjaia, Route Targua Ouzmour, 06000 Béjaia, Algeria

## Abstract

The mol­ecule of the title compound, C_23_H_23_ClN_2_O_4_, contains a quinolyl unit linked to a functionalized pyrrolidine system with a 3,4-*trans* arrangement of the substituents. The unit cell contains two stereoisomers that have the absolute stereochemistry 3*S*,4*R* and 3*R*,4*S*. The pyrrolidine ring adopts a twist conformation with pseudo-rotation parameters *P* = 258.2 (3)° and τ(*M*) = 35.3 (1)°. The packing is stabilized by C—H⋯π inter­actions and offset π–π stacking (centroid-to-centroid distance = 3.849 Å, inter­planar distance = 3.293 Å and slippage = 1.994 Å) between phenyl rings, leading to a two-dimensional network.

## Related literature

For general background, see: Padwa *et al.* (1999 ▶); Sahu *et al.* (2002 ▶); Robert & Meunier (1998 ▶); Dow *et al.* (2006 ▶); Witherup *et al.* (1995 ▶); Kravchenko *et al.* (2005 ▶); Bouraiou *et al.* (2008 ▶); Rezig *et al.* (2000 ▶); Moussaoui *et al.* (2002 ▶); Menasra *et al.* (2005 ▶); Rao *et al.* (1981 ▶). For related structures, see: Belfaitah *et al.* (2006 ▶); Bouraiou *et al.* (2007*a* ▶,*b* ▶).
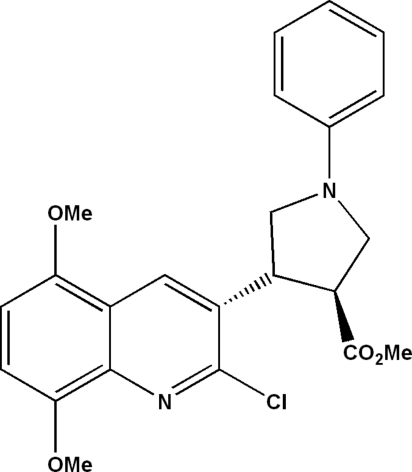


## Experimental

### 

#### Crystal data

C_23_H_23_ClN_2_O_4_
*M* *_r_* = 426.88
Monoclinic, 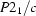

*a* = 9.579 (1) Å
*b* = 17.518 (1) Å
*c* = 12.944 (2) Å
β = 109.01 (2)°
*V* = 2053.6 (5) Å^3^
*Z* = 4
Mo *K*α radiation
μ = 0.22 mm^−1^
*T* = 296 (2) K
0.15 × 0.06 × 0.05 mm

#### Data collection

Nonius KappaCCD diffractometer
Absorption correction: none
9271 measured reflections
4717 independent reflections
3062 reflections with *I* > 2σ(*I*)
*R*_int_ = 0.026

#### Refinement

*R*[*F* ^2^ > 2σ(*F* ^2^)] = 0.055
*wR*(*F* ^2^) = 0.178
*S* = 1.03
4717 reflections
274 parameters
H-atom parameters constrained
Δρ_max_ = 0.38 e Å^−3^
Δρ_min_ = −0.33 e Å^−3^

### 

Data collection: *COLLECT* (Nonius, 1998 ▶); cell refinement: *SCALEPACK* (Otwinowski & Minor, 1997 ▶); data reduction: *DENZO* (Otwinowski & Minor, 1997 ▶) and *SCALEPACK*; program(s) used to solve structure: *SIR2002* (Burla *et al.*, 2003 ▶); program(s) used to refine structure: *SHELXL97* (Sheldrick, 2008 ▶); molecular graphics: *ORTEPIII* (Burnett & Johnson, 1996 ▶), *ORTEP-3 for Windows* (Farrugia, 1997 ▶) and *CAMERON* (Pearce *et al.*, 2000 ▶); software used to prepare material for publication: *PLATON* (Spek, 2003 ▶) and *WinGX* (Farrugia, 1999 ▶).

## Supplementary Material

Crystal structure: contains datablocks global, I. DOI: 10.1107/S1600536808031838/dn2384sup1.cif

Structure factors: contains datablocks I. DOI: 10.1107/S1600536808031838/dn2384Isup2.hkl

Additional supplementary materials:  crystallographic information; 3D view; checkCIF report

## Figures and Tables

**Table 1:** Hydrogen-bond geometry (Å, °) *Cg*1 is the centroid of the C17–C22 ring.

*D*—H⋯*A*	*D*—H	H⋯*A*	*D*⋯*A*	*D*—H⋯*A*
C12—H12*B*⋯*Cg*1^i^	0.96	2.67	3.601 (3)	164

Symmetry code: (i) 
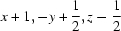
.

**Fig. 1.**
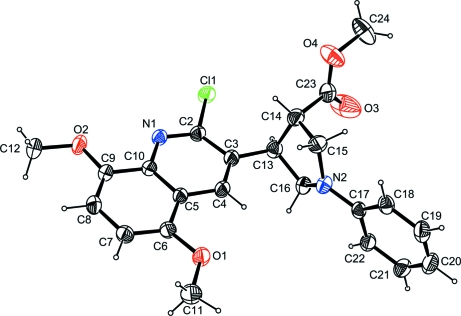
Molecular structure of the title compound with the atom-labeling scheme.Displacement ellipsoids are drawn at the 50% probability level. H atoms are represnted as small spheres of arbitrary radii.

**Fig. 2.**
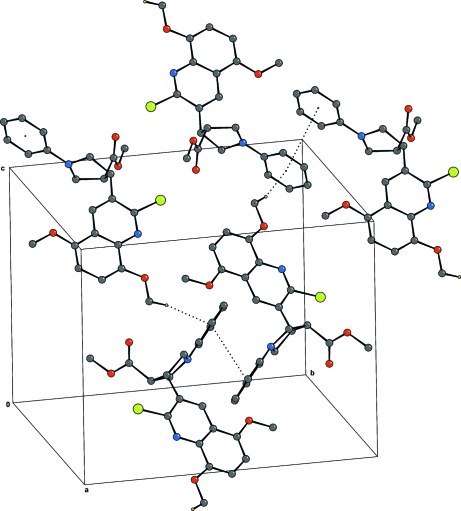
Partial packing view showing the C—H···π and π-π interactions drawn as dashed lines. H atoms not involved in H bonding interactions have been omitted for clarity.

## supplementary crystallographic information

### Comment

Quinolines derivatives have attracted considerable interest for many years due to their presence in the skeleton of a large number of bioactive compounds and natural products (Padwa *et al.*, 1999; Sahu *et al.* 2002). For example, quinoline alkaloids, such as quinine, chloroquin, mefloquine and amodiaquine, are used as efficient drugs for the treatment of malaria (Robert & Meunier, 1998; Dow *et al.*, 2006). On the other hand, pyrrolidine containing compounds are also of significant importance because of their biological activities and widespread employment in catalysis (Witherup *et al.*, 1995; Kravchenko *et al.*, 2005). As a part of our program related to the preparation and biological evaluation of quinolyl derivatives (Belfaitah *et al.*, 2006; Bouraiou *et al.*, 2008, 2007*a*,b; Rezig *et al.*, 2000; Moussaoui *et al.*, 2002), we have previously reported the preparation of some 3-pyrrolylquinoline derivatives *via* an 1,3-dipolar cycloaddition/oxydation key sequence from quinolinyl α,β-unsaturated esters as starting materials (Menasra *et al.*, 2005). In a continuation of our efforts in this area, we report here the crystal structure of new *N*-phenylpyrrolidine derivative bearing a quinoline ring at C-3 and ester group at C-4 *via* an 1,3-dipolar cycloaddition reaction.

The asymmetric unit of title compound contains a quinolyl unit linked to a functionalized pyrrolidine system with a 3,4-*trans* arrangement of the substituents (Fig. 1). The two rings of quinolyl moiety are fused in an axial fashion and form a dihedral angle of 0.67 (6)° and this quasi plane system forms dihedral angles of 83.76 (7)° with the phenyl ring.The pyrrolidine was obtained with conservation of the stereochemistry of starting alkene, giving only one diastereoisomer with no evidence of any other isomers in the ^1^H NMR spectra or thin-layer chromatography of the crude product. X-ray crystallography of (I) showed an asymmetric unit which contains only one stereoisomer and the analysis of the unit cell demonstrate that the second stereoisomer is generated via a symmetry element. The two stereoisomers have for each one, the absolute stereochemistry 3*S*,4*R* and 3*R*,4*S* of the new stereocenters created in the cycloaddition reactions.

The pyrrolidine ring adopts twist conformation on C13—C14 with pseudorotation parameters P = 258.2 (3)° and τ(*M*) = 35.3 (1)° (Rao *et al.*, 1981), the C13 atom deviates by 0.213 (2)Å from the mean plane through the remaining atoms.

The packing is stabilized by C—H···π interaction involving the C17—C22 phenyl ring (Table 1). Offset π···π stacking between this phenyl ring and the symmetry (-*x*, -*y*, -*z*) related ring might also be considered with a centroid-to-centroid distance of 3.849 Å, an interplanar distance of 3.293Å and a slippage of 1.994Å (Spek, 2003). These weak interactions build up a two dimensional network (Fig. 2).

### Experimental

The title compound I was synthesized by refluxing 1.0 mmol of (*E*)-methyl 3-(2-chloro-5,8-dimethoxyquinolin-3-yl) acrylate, 2.0 mmol of *N*-phenylglycine, and 5.0 mmol of CH_2_O in dry toluene (5.10^-3^*M*). The contents were then cooled and filtered off and the filtrate was concentrated under reduced pressure. The residue was subjected to column chromatography (silica gel, eluent: CH_2_Cl_2_) to afford pure product. Crystals suitable for X-ray analysis were obtained by slow evaporation of a dichloromethane solution of (I).

### Refinement

All H atoms were localized on Fourier maps but introduced in calculated positions and treated as riding on their parent C atom.

### Crystal data

**Table:**

C_23_H_23_ClN_2_O_4_	*F*(000) = 896
*M**_r_* = 426.88	*D*_x_ = 1.381 Mg m^−^^3^
Monoclinic, *P*2_1_/*c*	Mo *K*α radiation, λ = 0.71073 Å
Hall symbol: -P 2ybc	Cell parameters from 4717 reflections
*a* = 9.579 (1) Å	θ = 2.0–27.5°
*b* = 17.518 (1) Å	µ = 0.22 mm^−^^1^
*c* = 12.944 (2) Å	*T* = 296 K
β = 109.01 (2)°	Needle, white
*V* = 2053.6 (5) Å^3^	0.15 × 0.06 × 0.05 mm
*Z* = 4	

### Data collection

**Table:**

Nonius KappaCCD diffractometer	3062 reflections with *I* > 2σ(*I*)
Radiation source: fine-focus sealed tube	*R*_int_ = 0.026
graphite	θ_max_ = 27.5°, θ_min_ = 2.0°
φ scans, and ω scans with κ offsets	*h* = −12→12
9271 measured reflections	*k* = −22→22
4717 independent reflections	*l* = −16→16

### Refinement

**Table:**

Refinement on *F*^2^	Primary atom site location: structure-invariant direct methods
Least-squares matrix: full	Secondary atom site location: difference Fourier map
*R*[*F*^2^ > 2σ(*F*^2^)] = 0.055	Hydrogen site location: inferred from neighbouring sites
*wR*(*F*^2^) = 0.178	H-atom parameters constrained
*S* = 1.03	*w* = 1/[σ^2^(*F*_o_^2^) + (0.102*P*)^2^ + 0.336*P*] where *P* = (*F*_o_^2^ + 2*F*_c_^2^)/3
4717 reflections	(Δ/σ)_max_ < 0.001
274 parameters	Δρ_max_ = 0.38 e Å^−^^3^
0 restraints	Δρ_min_ = −0.33 e Å^−^^3^

### Special details

**Table:**

Geometry. All e.s.d.'s (except the e.s.d. in the dihedral angle between two l.s. planes) are estimated using the full covariance matrix. The cell e.s.d.'s are taken into account individually in the estimation of e.s.d.'s in distances, angles and torsion angles; correlations between e.s.d.'s in cell parameters are only used when they are defined by crystal symmetry. An approximate (isotropic) treatment of cell e.s.d.'s is used for estimating e.s.d.'s involving l.s. planes.
Refinement. Refinement of *F*^2^ against ALL reflections. The weighted *R*-factor *wR* and goodness of fit *S* are based on *F*^2^, conventional *R*-factors *R* are based on *F*, with *F* set to zero for negative *F*^2^. The threshold expression of *F*^2^ > σ(*F*^2^) is used only for calculating *R*-factors(gt) *etc*. and is not relevant to the choice of reflections for refinement. *R*-factors based on *F*^2^ are statistically about twice as large as those based on *F*, and *R*- factors based on ALL data will be even larger.

### Fractional atomic coordinates and isotropic or equivalent isotropic displacement parameters (Å^2^)

**Table:**

	*x*	*y*	*z*	*U*_iso_*/*U*_eq_	
C2	0.4396 (2)	0.31838 (12)	−0.07671 (19)	0.0457 (5)	
C3	0.3691 (3)	0.26052 (12)	−0.03567 (19)	0.0482 (5)	
C4	0.3895 (3)	0.18796 (12)	−0.0673 (2)	0.0494 (5)	
H4	0.3446	0.1473	−0.0443	0.059*	
C5	0.4777 (2)	0.17338 (11)	−0.13446 (18)	0.0417 (5)	
C6	0.5028 (3)	0.09863 (12)	−0.16864 (19)	0.0463 (5)	
C7	0.5869 (3)	0.08969 (13)	−0.2347 (2)	0.0514 (5)	
H7	0.6021	0.0411	−0.2580	0.062*	
C8	0.6515 (3)	0.15355 (14)	−0.2683 (2)	0.0526 (6)	
H8	0.7089	0.1461	−0.3131	0.063*	
C9	0.6317 (2)	0.22567 (13)	−0.23653 (18)	0.0465 (5)	
C10	0.5433 (2)	0.23684 (12)	−0.16778 (17)	0.0417 (5)	
C11	0.4590 (4)	−0.03500 (14)	−0.1605 (3)	0.0733 (8)	
H11A	0.4239	−0.0394	−0.2387	0.110*	
H11B	0.4054	−0.0697	−0.1300	0.110*	
H11C	0.5623	−0.0473	−0.1334	0.110*	
C12	0.7822 (3)	0.28219 (17)	−0.3307 (3)	0.0708 (8)	
H12A	0.8597	0.2464	−0.2973	0.106*	
H12B	0.8247	0.3308	−0.3377	0.106*	
H12C	0.7244	0.2640	−0.4017	0.106*	
C13	0.2718 (3)	0.27974 (13)	0.0337 (2)	0.0520 (6)	
H13	0.3102	0.3251	0.0781	0.062*	
C14	0.1144 (3)	0.29516 (14)	−0.0433 (2)	0.0559 (6)	
H14	0.1170	0.3162	−0.1129	0.067*	
C15	0.0444 (3)	0.21579 (13)	−0.0605 (2)	0.0548 (6)	
H15A	−0.0596	0.2188	−0.0682	0.066*	
H15B	0.0544	0.1921	−0.1255	0.066*	
C16	0.2513 (3)	0.21506 (13)	0.10654 (19)	0.0487 (5)	
H16A	0.3385	0.1830	0.1305	0.058*	
H16B	0.2314	0.2351	0.1702	0.058*	
C17	0.0583 (2)	0.11625 (12)	0.08000 (18)	0.0441 (5)	
C18	−0.0791 (3)	0.08503 (13)	0.0195 (2)	0.0500 (5)	
H18	−0.1277	0.1023	−0.0511	0.060*	
C19	−0.1420 (3)	0.02882 (15)	0.0646 (2)	0.0619 (7)	
H19	−0.2333	0.0089	0.0237	0.074*	
C20	−0.0738 (3)	0.00156 (15)	0.1677 (3)	0.0694 (8)	
H20	−0.1182	−0.0361	0.1971	0.083*	
C21	0.0624 (3)	0.03104 (15)	0.2276 (2)	0.0651 (7)	
H21	0.1104	0.0127	0.2976	0.078*	
C22	0.1281 (3)	0.08755 (13)	0.1845 (2)	0.0519 (6)	
H22	0.2199	0.1066	0.2259	0.062*	
C23	0.0291 (3)	0.34891 (17)	0.0089 (3)	0.0662 (7)	
C24	−0.1671 (4)	0.4343 (2)	−0.0293 (4)	0.1079 (13)	
H24A	−0.2517	0.4048	−0.0297	0.162*	
H24B	−0.1985	0.4769	−0.0781	0.162*	
H24C	−0.1176	0.4527	0.0433	0.162*	
Cl1	0.41733 (7)	0.41375 (3)	−0.04310 (5)	0.0582 (2)	
N1	0.5228 (2)	0.30920 (10)	−0.13716 (15)	0.0447 (4)	
N2	0.1249 (2)	0.17236 (11)	0.03698 (15)	0.0487 (5)	
O1	0.4375 (2)	0.04110 (9)	−0.13011 (16)	0.0635 (5)	
O2	0.6896 (2)	0.29035 (10)	−0.26382 (16)	0.0650 (5)	
O3	0.0479 (4)	0.3523 (2)	0.1042 (3)	0.1496 (14)	
O4	−0.0678 (3)	0.38737 (15)	−0.0644 (2)	0.0947 (8)	

### Atomic displacement parameters (Å^2^)

**Table:**

	*U*^11^	*U*^22^	*U*^33^	*U*^12^	*U*^13^	*U*^23^
C2	0.0513 (12)	0.0368 (10)	0.0521 (12)	−0.0025 (9)	0.0211 (10)	0.0011 (9)
C3	0.0518 (13)	0.0418 (11)	0.0572 (14)	−0.0015 (9)	0.0262 (11)	−0.0001 (9)
C4	0.0542 (13)	0.0390 (10)	0.0623 (14)	−0.0046 (9)	0.0292 (11)	0.0025 (10)
C5	0.0422 (11)	0.0385 (10)	0.0478 (12)	−0.0029 (8)	0.0192 (9)	0.0003 (8)
C6	0.0469 (12)	0.0387 (11)	0.0562 (13)	−0.0012 (9)	0.0210 (10)	−0.0010 (9)
C7	0.0578 (14)	0.0439 (11)	0.0571 (14)	0.0027 (10)	0.0249 (11)	−0.0048 (10)
C8	0.0566 (14)	0.0570 (13)	0.0535 (13)	0.0023 (11)	0.0306 (11)	0.0002 (10)
C9	0.0472 (12)	0.0485 (12)	0.0480 (12)	−0.0020 (9)	0.0215 (10)	0.0055 (9)
C10	0.0404 (11)	0.0412 (11)	0.0447 (12)	−0.0007 (8)	0.0155 (9)	0.0037 (8)
C11	0.088 (2)	0.0378 (12)	0.106 (2)	−0.0050 (13)	0.0492 (18)	−0.0099 (13)
C12	0.0718 (18)	0.0793 (18)	0.0783 (19)	0.0021 (14)	0.0476 (16)	0.0170 (15)
C13	0.0581 (14)	0.0469 (12)	0.0550 (14)	−0.0054 (10)	0.0239 (11)	−0.0039 (10)
C14	0.0670 (15)	0.0525 (13)	0.0547 (14)	0.0026 (11)	0.0288 (12)	0.0043 (11)
C15	0.0602 (14)	0.0527 (13)	0.0488 (13)	−0.0032 (11)	0.0142 (11)	0.0016 (10)
C16	0.0485 (12)	0.0512 (12)	0.0483 (12)	−0.0079 (10)	0.0183 (10)	−0.0035 (9)
C17	0.0476 (12)	0.0414 (10)	0.0514 (12)	−0.0012 (9)	0.0270 (10)	−0.0052 (9)
C18	0.0488 (13)	0.0509 (12)	0.0552 (13)	−0.0036 (10)	0.0238 (11)	−0.0106 (10)
C19	0.0585 (15)	0.0552 (13)	0.0839 (19)	−0.0132 (12)	0.0394 (14)	−0.0167 (13)
C20	0.081 (2)	0.0564 (15)	0.089 (2)	−0.0128 (14)	0.0530 (18)	0.0000 (14)
C21	0.0787 (18)	0.0627 (15)	0.0649 (16)	0.0021 (14)	0.0386 (14)	0.0115 (12)
C22	0.0537 (13)	0.0528 (13)	0.0548 (14)	−0.0039 (10)	0.0252 (11)	0.0017 (10)
C23	0.0716 (17)	0.0722 (17)	0.0677 (18)	0.0082 (14)	0.0403 (15)	0.0035 (14)
C24	0.075 (2)	0.119 (3)	0.134 (3)	0.026 (2)	0.040 (2)	−0.042 (3)
Cl1	0.0772 (4)	0.0366 (3)	0.0688 (4)	−0.0023 (3)	0.0349 (3)	−0.0035 (2)
N1	0.0473 (10)	0.0404 (9)	0.0491 (10)	−0.0048 (8)	0.0194 (8)	0.0008 (7)
N2	0.0488 (10)	0.0526 (11)	0.0449 (10)	−0.0100 (8)	0.0153 (8)	0.0012 (8)
O1	0.0766 (12)	0.0357 (8)	0.0950 (14)	−0.0065 (8)	0.0510 (11)	−0.0048 (8)
O2	0.0799 (12)	0.0537 (10)	0.0820 (13)	−0.0051 (9)	0.0546 (11)	0.0084 (8)
O3	0.196 (3)	0.168 (3)	0.098 (2)	0.098 (3)	0.065 (2)	0.008 (2)
O4	0.0841 (15)	0.1037 (17)	0.0949 (17)	0.0388 (13)	0.0272 (13)	−0.0179 (13)

### Geometric parameters (Å, °)

**Table:**

C2—N1	1.296 (3)	C13—H13	0.9800
C2—C3	1.414 (3)	C14—C15	1.528 (3)
C2—Cl1	1.757 (2)	C14—C23	1.540 (4)
C3—C4	1.369 (3)	C14—H14	0.9800
C3—C13	1.528 (3)	C15—N2	1.460 (3)
C4—C5	1.419 (3)	C15—H15A	0.9700
C4—H4	0.9300	C15—H15B	0.9700
C5—C10	1.412 (3)	C16—N2	1.458 (3)
C5—C6	1.427 (3)	C16—H16A	0.9700
C6—C7	1.362 (3)	C16—H16B	0.9700
C6—O1	1.363 (3)	C17—N2	1.384 (3)
C7—C8	1.413 (3)	C17—C22	1.393 (3)
C7—H7	0.9300	C17—C18	1.405 (3)
C8—C9	1.361 (3)	C18—C19	1.379 (3)
C8—H8	0.9300	C18—H18	0.9300
C9—O2	1.358 (3)	C19—C20	1.368 (4)
C9—C10	1.427 (3)	C19—H19	0.9300
C10—N1	1.361 (3)	C20—C21	1.382 (4)
C11—O1	1.424 (3)	C20—H20	0.9300
C11—H11A	0.9600	C21—C22	1.384 (3)
C11—H11B	0.9600	C21—H21	0.9300
C11—H11C	0.9600	C22—H22	0.9300
C12—O2	1.434 (3)	C23—O3	1.190 (4)
C12—H12A	0.9600	C23—O4	1.281 (4)
C12—H12B	0.9600	C24—O4	1.439 (3)
C12—H12C	0.9600	C24—H24A	0.9600
C13—C16	1.527 (3)	C24—H24B	0.9600
C13—C14	1.536 (4)	C24—H24C	0.9600
			
N1—C2—C3	126.9 (2)	C13—C14—H14	110.4
N1—C2—Cl1	114.55 (15)	C23—C14—H14	110.4
C3—C2—Cl1	118.52 (17)	N2—C15—C14	105.40 (19)
C4—C3—C2	114.9 (2)	N2—C15—H15A	110.7
C4—C3—C13	123.70 (19)	C14—C15—H15A	110.7
C2—C3—C13	121.36 (19)	N2—C15—H15B	110.7
C3—C4—C5	121.5 (2)	C14—C15—H15B	110.7
C3—C4—H4	119.3	H15A—C15—H15B	108.8
C5—C4—H4	119.3	N2—C16—C13	104.32 (19)
C10—C5—C4	117.30 (19)	N2—C16—H16A	110.9
C10—C5—C6	119.41 (18)	C13—C16—H16A	110.9
C4—C5—C6	123.29 (18)	N2—C16—H16B	110.9
C7—C6—O1	125.5 (2)	C13—C16—H16B	110.9
C7—C6—C5	119.60 (19)	H16A—C16—H16B	108.9
O1—C6—C5	114.91 (18)	N2—C17—C22	120.6 (2)
C6—C7—C8	120.6 (2)	N2—C17—C18	121.5 (2)
C6—C7—H7	119.7	C22—C17—C18	117.8 (2)
C8—C7—H7	119.7	C19—C18—C17	120.1 (2)
C9—C8—C7	121.6 (2)	C19—C18—H18	120.0
C9—C8—H8	119.2	C17—C18—H18	120.0
C7—C8—H8	119.2	C20—C19—C18	121.8 (3)
O2—C9—C8	126.0 (2)	C20—C19—H19	119.1
O2—C9—C10	115.04 (19)	C18—C19—H19	119.1
C8—C9—C10	118.99 (19)	C19—C20—C21	118.7 (2)
N1—C10—C5	121.56 (18)	C19—C20—H20	120.6
N1—C10—C9	118.70 (18)	C21—C20—H20	120.6
C5—C10—C9	119.73 (19)	C20—C21—C22	120.8 (3)
O1—C11—H11A	109.5	C20—C21—H21	119.6
O1—C11—H11B	109.5	C22—C21—H21	119.6
H11A—C11—H11B	109.5	C21—C22—C17	120.8 (2)
O1—C11—H11C	109.5	C21—C22—H22	119.6
H11A—C11—H11C	109.5	C17—C22—H22	119.6
H11B—C11—H11C	109.5	O3—C23—O4	124.8 (3)
O2—C12—H12A	109.5	O3—C23—C14	124.3 (3)
O2—C12—H12B	109.5	O4—C23—C14	110.9 (2)
H12A—C12—H12B	109.5	O4—C24—H24A	109.5
O2—C12—H12C	109.5	O4—C24—H24B	109.5
H12A—C12—H12C	109.5	H24A—C24—H24B	109.5
H12B—C12—H12C	109.5	O4—C24—H24C	109.5
C16—C13—C3	115.15 (19)	H24A—C24—H24C	109.5
C16—C13—C14	103.61 (19)	H24B—C24—H24C	109.5
C3—C13—C14	108.3 (2)	C2—N1—C10	117.82 (18)
C16—C13—H13	109.8	C17—N2—C16	120.98 (18)
C3—C13—H13	109.8	C17—N2—C15	122.38 (19)
C14—C13—H13	109.8	C16—N2—C15	111.38 (18)
C15—C14—C13	103.03 (19)	C6—O1—C11	117.75 (19)
C15—C14—C23	110.5 (2)	C9—O2—C12	117.3 (2)
C13—C14—C23	111.8 (2)	C23—O4—C24	117.5 (3)
C15—C14—H14	110.4		

### Hydrogen-bond geometry (Å, °)

**Table:**

*D*—H···*A*	*D*—H	H···*A*	*D*···*A*	*D*—H···*A*
C12—H12B···Cg1^i^	0.96	2.67	3.601 (3)	164

Symmetry codes: (i) *x*+1, −*y*+1/2, *z*−1/2.
